# Gelsolin Plays a Role in the Actin Polymerization Complex of Hair Cell Stereocilia

**DOI:** 10.1371/journal.pone.0011627

**Published:** 2010-07-16

**Authors:** Philomena Mburu, María Rosario Romero, Helen Hilton, Andrew Parker, Stuart Townsend, Yoshiaki Kikkawa, Steve D. M. Brown

**Affiliations:** 1 Medical Research Council Mammalian Genetics Unit, Harwell Science and Innovation Campus, Oxfordshire, United Kingdom; 2 Department of Bioproduction, Tokyo University of Agriculture, Abashiri, Japan; University of Birmingham, United Kingdom

## Abstract

A complex of proteins scaffolded by the PDZ protein, whirlin, reside at the stereocilia tip and are critical for stereocilia development and elongation. We have shown that in outer hair cells (OHCs) whirlin is part of a larger complex involving the MAGUK protein, p55, and protein 4.1R. Whirlin interacts with p55 which is expressed exclusively in outer hair cells (OHC) in both the long stereocilia that make up the stereocilia bundle proper as well as surrounding shorter microvilli that will eventually regress. In erythrocytes, p55 forms a tripartite complex with protein 4.1R and glycophorin C promoting the assembly of actin filaments and the interaction of whirlin with p55 indicates that it plays a similar role in OHC stereocilia. However, the components directly involved in actin filament regulation in stereocilia are unknown. We have investigated additional components of the whirlin interactome by identifying interacting partners to p55. We show that the actin capping and severing protein, gelsolin, is a part of the whirlin complex. Gelsolin is detected in OHC where it localizes to the tips of the shorter rows but not to the longest row of stereocilia and the pattern of localisation at the apical hair cell surface is strikingly similar to p55. Like p55, gelsolin is ablated in the *whirler* and *shaker2* mutants. Moreover, in a gelsolin mutant, stereocilia in the apex of the cochlea become long and straggly indicating defects in the regulation of stereocilia elongation. The identification of gelsolin provides for the first time a link between the whirlin scaffolding protein complex involved in stereocilia elongation and a known actin regulatory molecule.

## Introduction

Stereocilia, actin-filled structures on the surface of hair cells, are vital for the process of mechanoelectrical transduction in the auditory and vestibular systems. Stereocilia are organized into bundles whose extraordinary feature is a regular staircase pattern. The bundles consist of several rows of stereocilia that are ordered according to height. Stereocilia develop from microvilli on the surface of hair cells in the region of the kinocilium [1]–[3]. Little is known of the molecular processes of stereocilia development and how the exquisite staircase structure of the stereocilia bundle is determined. A mutation in the whirlin gene (*Whrn^wi^*) underlies the *whirler* deafness mutant [4]. The *whirler* mutation is characterised by shortened stereocilia, identifying a key function for the PDZ protein, whirlin, in stereocilia growth and actin polymerisation. Whirlin is expressed at the stereocilia tip [5]–[7]. The *shaker2* mutant carries a mutation in the myosin XVa gene (*Myo15a^sh2^*) and similar to the *whirler* mutant shows short stereocilia [8]. Myosin XVa also localizes to the stereocilia tip and like whirlin appears essential for stereocilia elongation [9]. Whirlin has been shown to interact with myosin XVa via its third PDZ domain [5], [6] and myosin XVA mutants fail to localize whirlin at the stereocilia tip [5]. It appears that myosin XVa is required for delivery of whirlin to the stereocilia tip where it appears to act as a scaffolding protein for organizing a protein complex controlling actin polymerization and stereocilia organization [5], [10].

We have shown that in outer hair cells (OHCs) whirlin is part of a larger complex involving the MAGUK protein, p55, and protein 4.1R [11]. Whirlin interacts with p55 which is expressed exclusively in outer hair cells (OHC) in both the long stereocilia that make up the stereocilia bundle proper as well as the shorter microvilli that will eventually regress. p55 interacts with protein 4.1R in erythrocytes [12], and 4.1R is also expressed in stereocilia with an identical pattern to p55. Mutations in both whirlin and myosin XVa lead to early ablation of p55 and 4.1R labeling of stereocilia. In erythrocytes, p55 forms a tripartite complex with protein 4.1R and the cell-surface molecule, glycophorin C promoting the assembly of actin/spectrin filaments [12], and the interaction of whirlin with p55 indicates that it plays a similar role in OHC stereocilia.

Overall, the data indicate that whirlin is a critical scaffolding molecule for the assembly of a protein complex at the stereocilia tip governing actin polymerization and stereocilia elongation. We have searched for additional members of this complex, in particular to identify regulatory molecules that might be key to the control of actin polymerization.

## Results

### Interaction of the proteins p55 and gelsolin

**Figure 1:**
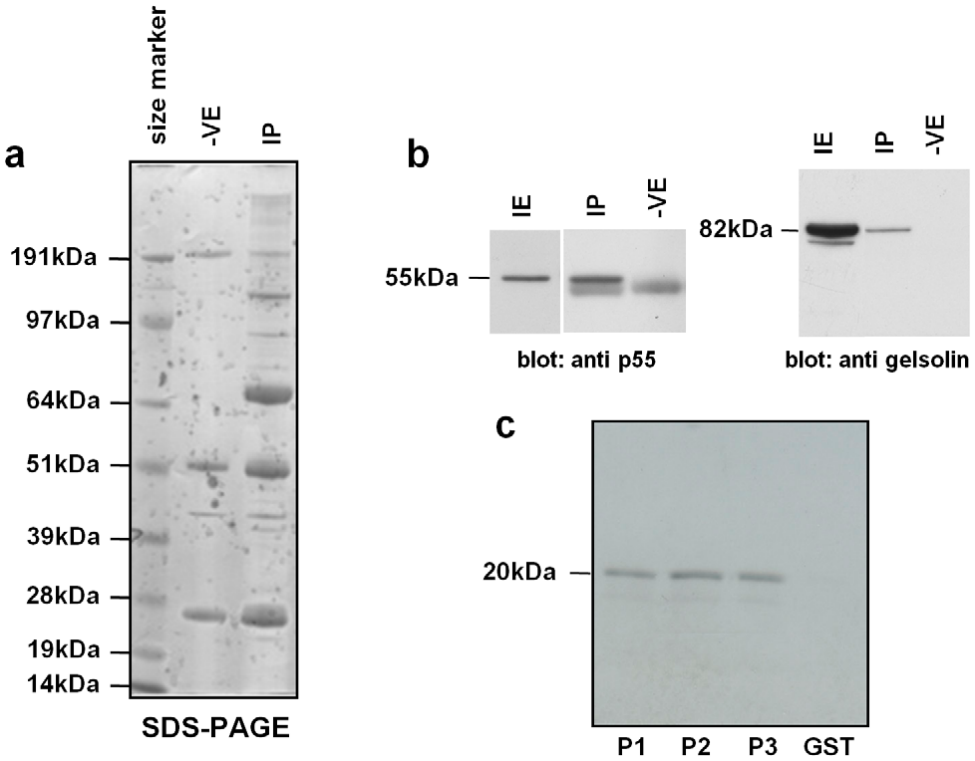
*In vivo* and *in vitro* interactions of p55 and gelsolin. **a. SDS-PAGE analyses of proteins immunoprecipitated from inner ear lysates.** SDS-PAGE showing proteins immunoprecipitated from inner ear lysates with anti p55 antibody or with normal goat IgG as a negative control. Several bands can be observed that are present in the IP lane and absent from the negative lane. LC-MS/MS analysis of eluted IP complexes identified p55 and gelsolin, among other proteins, only in the p55 IP and not in the negative control, suggesting that they are interacting partners. **b. Western blot analysis of immunoprecipitates.** Western blot of anti p55 antibody immunoprecipitates showing co-IP of gelsolin. Proteins immunoprecipitated from inner ear lysates with anti p55 antibody (IP) or with normal goat IgG as a negative control (-VE) were analysed by western blotting with either anti p55 antibody (upper panel) or anti gelsolin antibody (lower panel) IE: inner ear lysate. **c. *In vitro* pull down assays confirmed the interaction of gelsolin with p55.** Three GST-tagged gelsolin constructs containing either S1 and S2 (P1), S3 and S4 (P2) or S5 and S6 (P3) gelsolin domains were expressed in E. coli K12 ER2508 cells using pGEX-4T1. Immobilised protein was incubated with *in vitro* translated ^35^S-labelled GUK domain of p55. GST protein alone was used as a negative control.

We set out to explore interacting partners to the protein p55 that had already been identified as a component of the whirlin complex [11]. Immunoprecipitations (IPs) from inner ear lysate using a p55 antibody followed by liquid chromatography tandem mass spectrometry (LC-MS/MS) (Fig. 1a) identified a number of putative interacting partners, including gelsolin. We confirmed the interaction between p55 and gelsolin both *in vivo* and *in vitro*. Firstly, p55 immunoprecipitations of inner ear lysates followed by western blotting with anti-gelsolin antibody demonstrated a specific interaction between p55 and gelsolin and indicated that the two proteins are part of the same complex in the inner ear (Fig. 1b). The guanylate kinase (GUK)-like domain of p55 lacks catalytic activity and is a protein-binding module [13], [14]. We therefore explored interactions *in vitro* between the p55 GUK domain and various regions of gelsolin. We found that GST fusions with the S1/S2, the S3/S4 and S5/S6 domains of gelsolin pulled down the p55 GUK domain (Fig. 1c).

### Localisation of gelsolin in cochlear and vestibular hair cells

**Figure 2:**
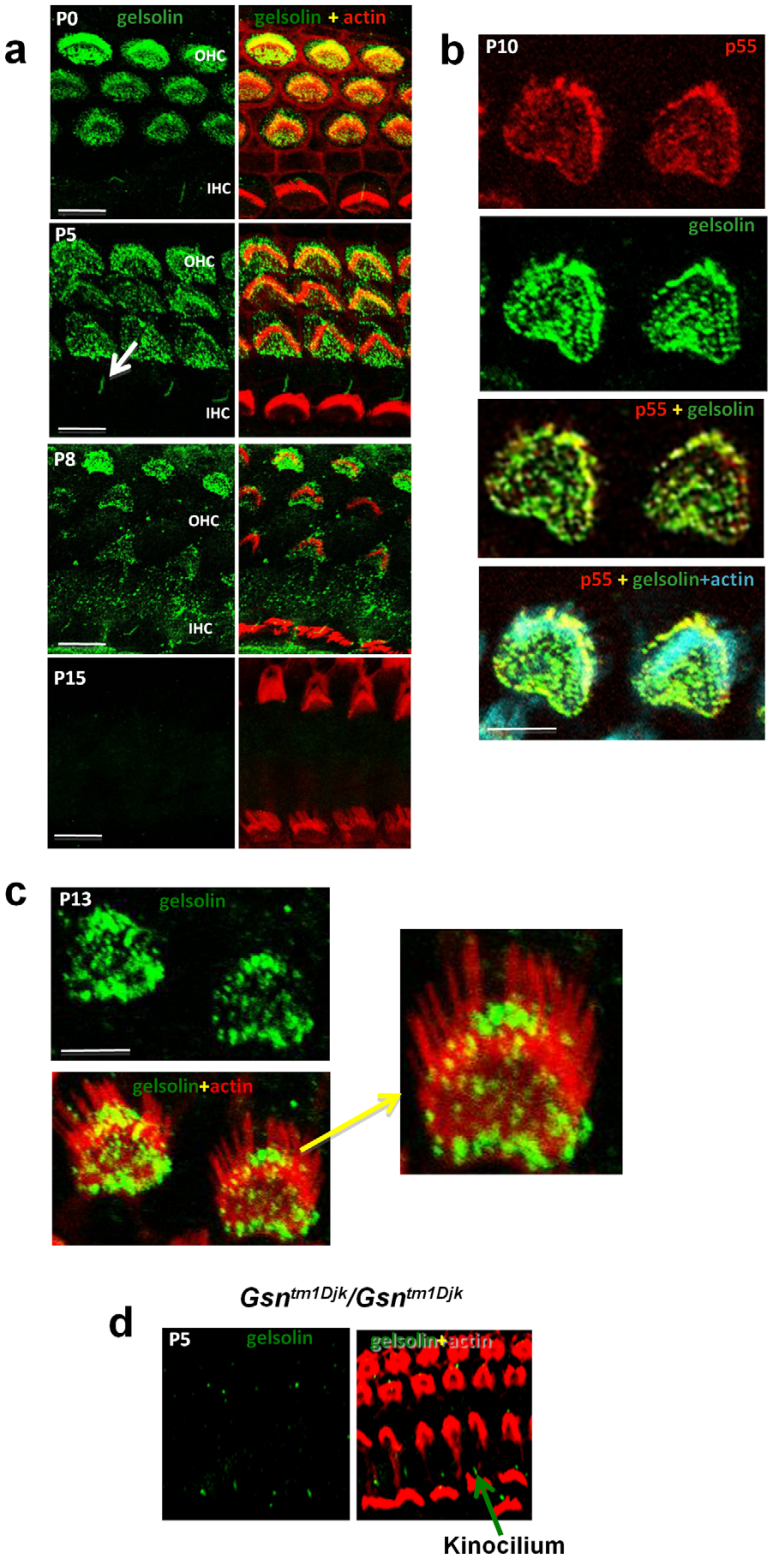
Gelsolin is localised to outer hair cell stereocilia where it co-localises with p55. **a. Gelsolin expression in outer hair cells.** Cochlear whole mounts were stained with antibody to gelsolin (green) and phalloidin (red) to detect actin; right hand panels show merge of gelsolin and actin. At time points P0 to P8, gelsolin is localized to stereocilia. Gelsolin is also detected outside of the stereocilia bundle in strial and neural domains of the apical hair cell surface, similar to p55 localisation. Labeling with gelsolin is weaker by P8 and has faded out by P15. Note absence of inner hair cell labeling. Arrow marks non-specific labeling of kinocilia in inner hair cell row, see also Fig. 2d (Scale bar, 5 µm). **b. Colocalisation of gelsolin and p55.** Cochlear whole mounts from postnatal day 10 (P10) mice stained with p55 (red), gelsolin (green) and actin (blue) (Scale bar, 5 µm). **c.** Gelsolin localises to the shorter rows and not to the longest row of stereocilia of the outer hair cells. Cochlear whole mounts from postnatal day 13 (P13) mice. Gelsolin (green) and actin (red). (Scale bar, 5 µm). **d.** Gelsolin is absent in hair bundles of the gelsolin knockout mouse (*Gsn^tm1Djk^/Gsn^tm1Djk^*) but is seen in the kinocilium demonstrating that this represents non-specific staining. Cochlear whole mounts from postnatal day 5 (P5) mice were stained with antibody to gelsolin (green) and phalloidin (red) to detect actin.

Given the observed interactions between p55 and gelsolin we undertook localisation studies of gelsolin in the stereocilia bundle and at the apical hair cell surface. Confocal studies using a gelsolin antibody localized the protein to the stereocilia bundle of OHCs along the entire length of the cochlear duct (Fig. 2a). We confirmed the specificity of the gelsolin antibody by demonstrating an absence of labelling in the outer hair cells of a gelsolin knockout mouse previously created – *Gsn^tm1Djk^/Gsn^tm1Djk^* [15] (Figure 2d). Gelsolin antibodies label the kinocilia that are present at the apex of hair cell bundles in both OHC and inner hair cell (IHC) rows (Fig. 2). However, this localization appears to be non-specific labeling as it is also seen in the gelsolin knockout mouse (Figure 2d). Gelsolin is confined to the outer hair cells and, like p55, is not expressed in inner hair cells (IHCs). Gelsolin is seen in OHCs from P0 till after P8 and expression fades out by P15 (Fig. 2a). Examination of 1 month old mice did not show any gelsolin labeling of OHCs. Stereocilia maturation is considered complete by around P7 [16] and thus gelsolin is present in the stereocilia bundle during the critical period of stereocilia development and elongation.

The pattern of gelsolin at the apical hair cell surface strikingly mimics the previously reported localization of p55 [11]. In addition, like p55, gelsolin shows significant labeling at the apical hair cell surface outside of the longest stereocilia (see Fig. 2a and 2b), both strial and neural to the stereocilia bundle proper. Double labeling with p55 and gelsolin antibodies shows strong co-localisation in both the stereocilia bundle and at the apical hair cell surface (Fig. 2b) supporting the interaction between these two proteins. Further we examined the localisation of gelsolin across the stereocilia bundle rows. It appears that gelsolin is confined to the shorter stereocilia rows and we did not observe any labeling of the longest row of stereocilia (Figure 2c). Finally, we investigated the expression of gelsolin in the vestibular system and found that gelsolin was absent in vestibular hair cells (Supplementary Information Figure S2).

### Functional analysis of gelsolin in stereocilia using *whirler* and *shaker2* mutants

**Figure 3:**
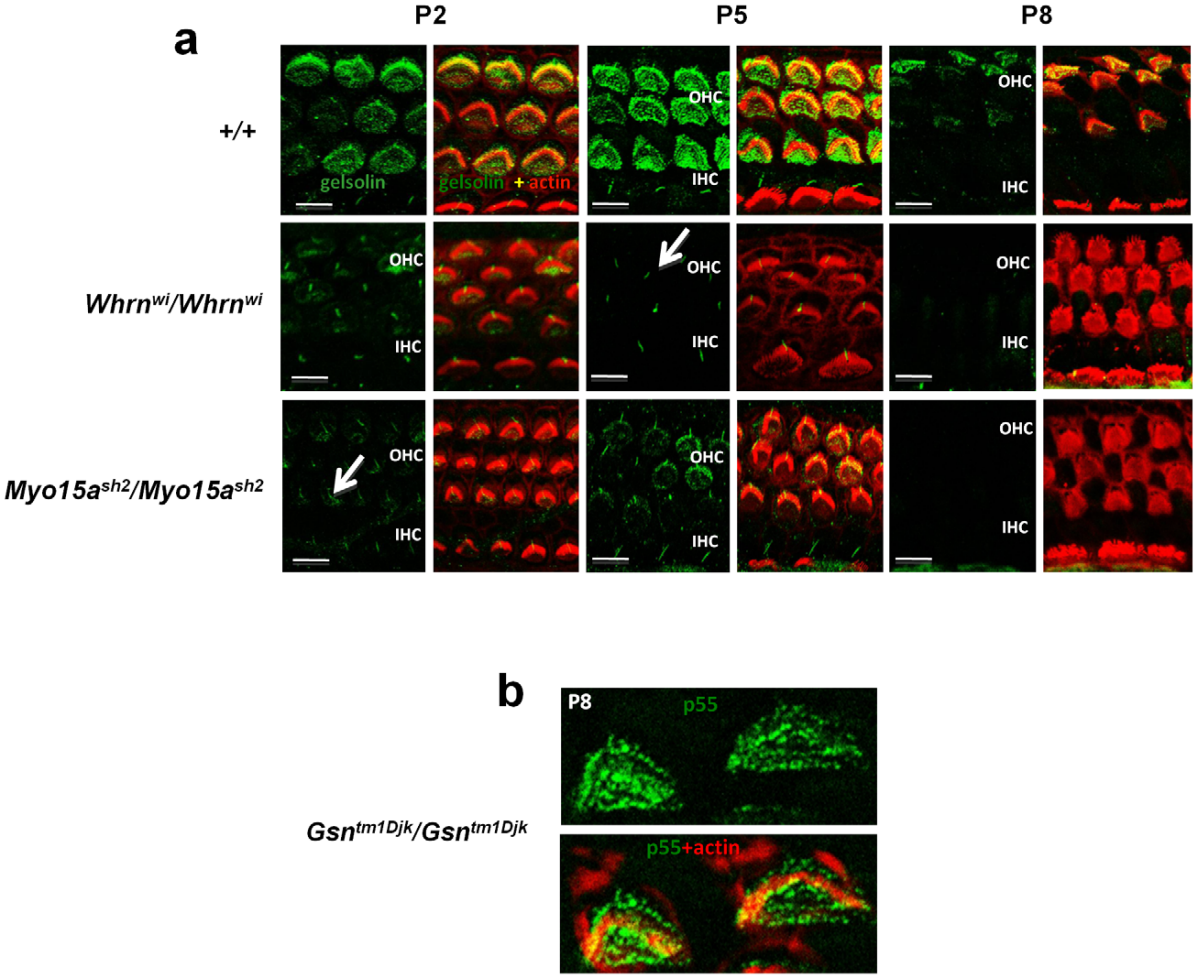
Ablation of gelsolin in mutant mice. **a. Ablation of gelsolin in whirler (** ***Whrn^wi^*** **) and shaker2 (** ***Myo15a^sh2^*** **) mutants.** Gelsolin expression (green) examined in wild-type, *Whrn^wi^/Whrn^wi^* and *Myo15a^sh2^/Myo15a^sh2^* mice (actin, red) at P2, P5 and P8. Expression of gelsolin is markedly reduced in both whirler and shaker2 mutants at P2 and P5, and completely ablated by P8. Note that labeling of kinocilia is unaffected, and the labeling of outer hair cell kinocilia is revealed, marked by arrow. (Scale bar, 5 µm). **b.** **p55 expression is not affected in the gelsolin knockout mouse (** ***Gsn^tm1Djk^/Gsn^tm1Djk^*** **).** Cochlear whole mounts from knockout mice (P8) were stained with antibody to p55 (green) and phalloidin (red) to detect actin. p55 expression was not affected. Similar results were seen at P4 (data not shown).

The interaction of gelsolin in the whirlin complex was confirmed and studied by investigating the localization of gelsolin in the *whirler* mutant (*Whrn^wi^*), which lacks whirlin, and also the *shaker2* mutant (*Myo15a^sh2^*), which lacks myosin XVa (Fig. 3). In both of these mutants we had already demonstrated that expression of p55 showed early ablation [11]. In the whirler mutant, p55 is ablated in the absence of whirlin from P5 onwards. Expression of p55 is completely ablated in *shaker2* mutant hair cells at all time points. We therefore sought to investigate the effects of the *whirler* and *shaker2* mutants on the localization of gelsolin in hair cells. In both the *whirler* and *shaker2* mutants, expression of gelsolin is considerably reduced at P2 and is ablated from P5, similar to that seen for p55 [11] (Fig. 3). Taken together the biochemical, localization and mutant studies indicate that gelsolin is part of the whirlin complex. We also investigated given the interaction of p55 and gelsolin, whether or not the localisation of p55 is affected in a gelsolin knock-out mouse (*Gsn^tm1Djk^/Gsn^tm1Djk^*) [15]. We found that the localization of p55 was unaffected (Figure 3b).

### Analysis of stereocilia organisation in a gelsolin mutant

We proceeded to explore the function of gelsolin in stereocilia by analyzing the gelsolin knockout mouse [15]. Gelsolin homozygous mutant mice have normal embryonic development and longevity. We proceeded to investigate the cochlear phenotype of gelsolin mutant mice focusing on the development of the stereocilia bundle.

**Figure 4:**
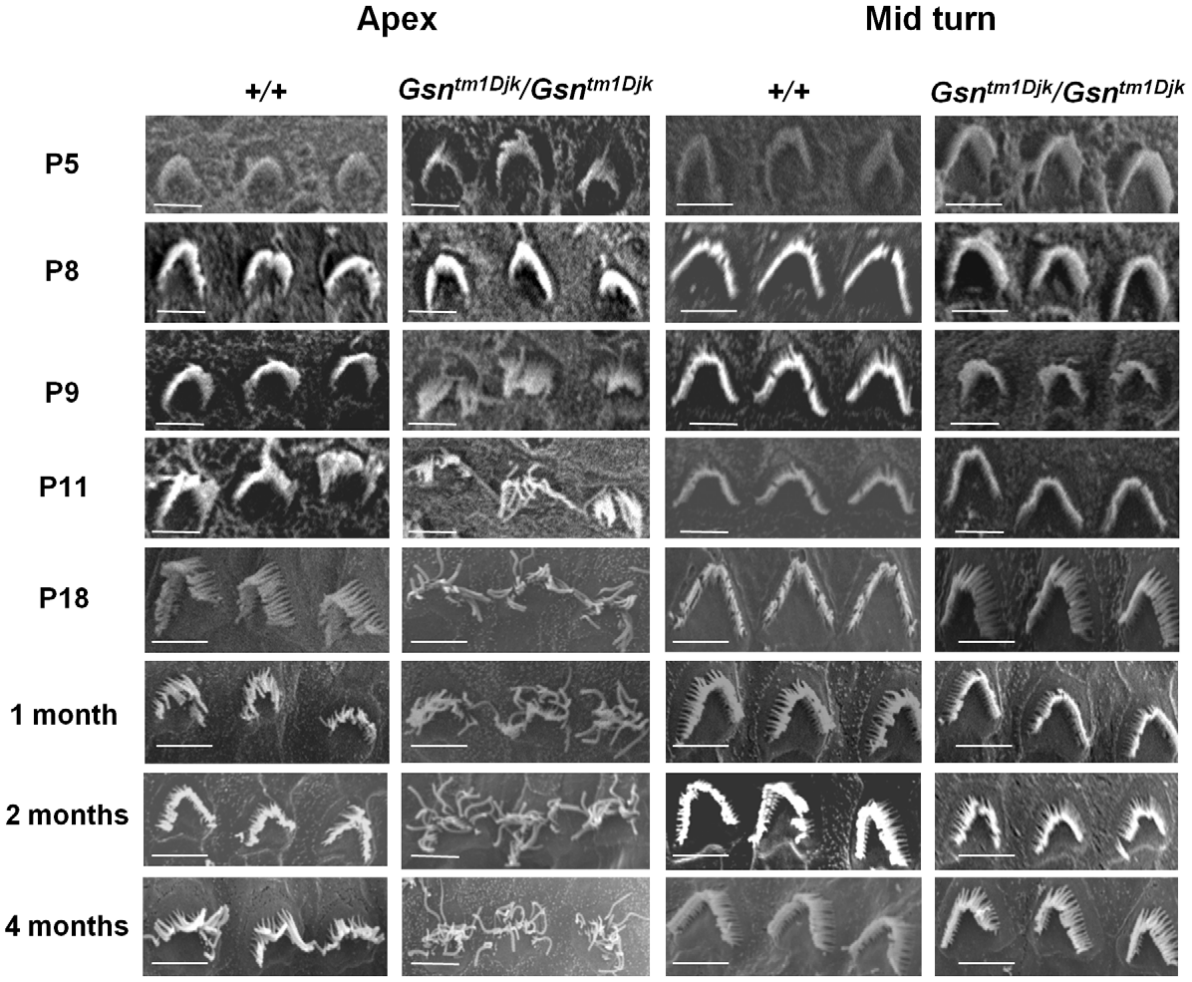
Ultrastructural analysis of cochlear outer hair cell stereocilia in gelsolin mutant mice. Analysis of outer hair cell morphology from P5 to 4 months of age in apical and middle turns in wild-type and gelsolin homozygous knock-out mice (*Gsn^tm1Djk^/Gsn^tm1Djk^*). Note from P9 stereocilia and bundle morphology in apical turn outer hair cells is abnormal with long thread-like stereocilia appearing from P11. (Scale bar, 10 µm).

SEM analysis of OHCs from gelsolin homozygous mutants showed abnormal OHC stereocilia shortly following stereocilia maturity. In apical outer hair cells the stereocilia bundle developed normally till P8. However from P9 the stereocilia bundle morphology began to show signs of disorganisation and from P11 the stereocilia bundle became completely disorganized with the stereocilia elongated and straggly (Fig. 4). We found that stereocilia defects were confined to the apex of the cochlea and no stereocilia dysmorphology was observed in the middle (Fig. 4) or basal regions (Supplementary Information Figure S1a). This is despite the widespread expression of gelsolin in stereocilia along the length of cochlear duct. IHC stereocilia morphology also appeared normal throughout the cochlea in the mutant (Supplementary Information Figure S1b) consistent with the absence of gelsolin in IHCs. We also assessed auditory responses in gelsolin homozygous mutants. We carried out auditory brainstem response (ABR) analysis in 2 month old mice but did not observe any significant hearing loss across all frequencies tested (Supplementary Information Figure S3). However, we would not expect the morphological effects of the gelsolin mutation, confined to stereocilia in the apical OHCs, to markedly affect hearing sensitivity. SEM analysis of the vestibular hair cells showed no stereocilia abnormality which is supported by the observation that gelsolin is not expressed in vestibular hair cells (Supplementary Information Figure S2).

### Epistasis of the whirler and gelsolin mutants

**Figure 5:**
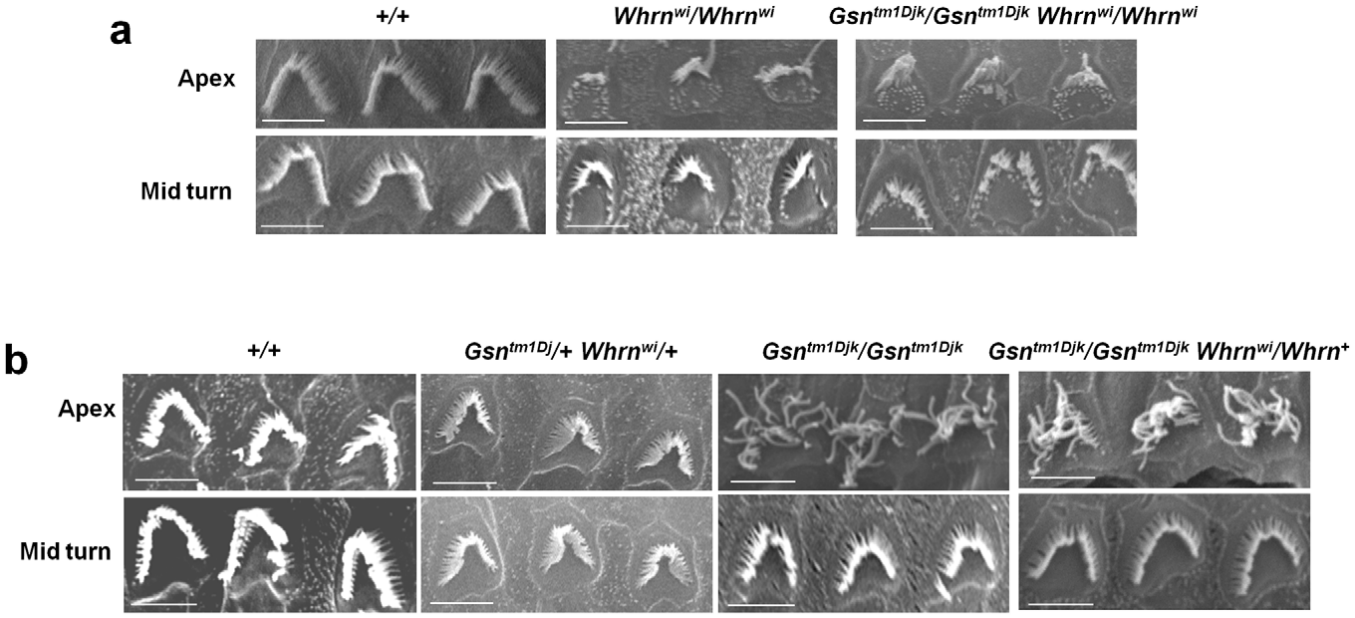
Ultrastructural analysis of cochlear outer hair cell stereocilia in gelsolin/whirler double mutant mice. **a. Outer hair cell morphology from apical turns in double mutant mice.** Analysis of outer hair cell morphology from apical turns in double mutant homozygotes for gelsolin and whirlin (*Gsn^tm1Djk^/Gsn^tm1Djk^ Whrn^wi^/Whrn^wi^*) compared to whirlin homozygotes (*Whrn^wi^/Whrn^wi^*) at 3 months of age. Note that the double mutants show the typical short stereocilia phenotype of the whirler mutant observed particularly towards the outer edges of the stereocilia bundle [17], and do not show the long and straggly stereocilia typical of the gelsolin mutant. (Scale bar, 10 µm). **b. Outer hair cell morphology from apical turns in double heterozygous mice.** Analysis of outer hair cell morphology from the apex and the middle of the cochlear turn of double heterozygous (*Gsn^tm1Djk^/+ Whrn^wi^/+)* and *Gsn^tm1Djk^/Gsn^tm1Djk^ Whrn^wi^/+* mice at 3 months of age. Double heterozygous mice demonstrate a wild type morphology, whereas *Gsn^tm1Djk^/Gsn^tm1Djk^ Whrn^wi^/+* mice show the typical long and straggly stereocilia seen in *Gsn^tm1Djk^/Gsn^tm1Djk^* mice (Scale bar, 10 µm).

The evidence that gelsolin is a part of the whirlin complex and affects stereocilia elongation led us to surmise that it plays a role in regulating actin polymerization, and that in the absence of gelsolin the whirlin interactome is still formed, actin polymerization proceeds but it is uncontrolled. Our hypothesis predicts that in the absence of whirlin, the whirlin/p55/4.1R/gelsolin complex would not be formed and that the *whirler* mutant would be epistatic to gelsolin, with double mutants displaying a shortened stereocilia phenotype and an absence of the elongated stereocilia phenotype displayed in gelsolin mutants. We therefore examined double mutant gelsolin/whirler homozygotes. We found that they display the short stereocilia phenotype (Fig. 5a) typical of the *whirler* mutant. As previously reported, the *whirler* phenotype in apical hair cells shows an irregular mixture of stereocilia of variable length with short stereocilia often observed towards the outer edges of the stereocilia bundle [17]. There is no evidence of the stereocilia phenotype seen in the apex of the cochlea of gelsolin mutants. We also examined the outer hair cell morphology in the apex and middle of the cochlear turn of both double heterozygotes (*Gsn^tm1Djk^/+ Whrn^wi^/+*) and *Gsn^tm1Djk^/Gsn^tm1Djk^ Whrn^wi^/+* mice. Both whirlin and gelsolin mutants are recessive and thus for the double heterozygotes we would predict a wild type phenotype, which is indeed observed (Fig. 5b). For *Gsn^tm1Djk^/Gsn^tm1Djk^ Whrn^wi^/+* mice we would predict a phenotype that corresponds to *Gsn^tm1Djk^/Gsn^tm1Djk^* mice, and again this is observed (Figure 5b). In conclusion, our observations on a number of combinations of double mutants support our hypothesis that the whirlin complex provides a scaffold for gelsolin that appropriately localized plays a role in controlling stereocilia elongation in the apical turns of the cochlea.

## Discussion

In erythrocytes, p55 is part of a larger complex incorporating protein 4.1R and the transmembrane protein, glycophorin C, that regulates actin polymerisation and cytoskeletal organisation. Our previous findings that p55 is a member of the whirlin complex indicated that p55 plays a similar role in hair cell stereocilia [11]. In order to elaborate the mechanisms by which p55 regulates actin polymerisation in the stereocilia we set out to explore further the nature of the whirlin complex by searching for additional interacting partners to p55. We reasoned that the identification of additional members of the whirlin complex may uncover key regulatory molecules involved in actin polymerization. Biochemical, ultrastructural and genetic data presented here demonstrate that gelsolin is a member of the whirlin complex and plays a role in stereocilia elongation.

Gelsolin is an actin-binding and severing protein that regulates both actin assembly and disassembly [18], [19]. It belongs to a group of actin-binding proteins that are composed of gelsolin-like repeats. Gelsolin is composed of six of these repeats (S1-6) that in the absence of calcium form a compact folded structure with the tail helix in close proximity to the actin binding helix of S2 – the so-called “tail latch”. Current models indicate that Ca^2+^ binding to S6 induces a conformational change releasing the tail latch and allowing binding of actin to S2. S2 binding induces the actin binding of S1 and severing and capping follow. Phosphatidylinositol-4,5-bisphosphate (PIP_2_) dissociates gelsolin from actin and allows actin elongation. The interaction of the p55 GUK domain with several gelsolin constructs suggests that the GUK domain binds directly to a motif common to the gelsolin repeats.

Our studies of the *whirler* and *shaker2* mutants indicate that gelsolin functions as a component of the whirlin complex. Whirlin appears to act as a scaffold for a number of proteins such as p55 and 4.1R, which as indicated above are involved in other cell types in actin polymerisation and cytoskeletal reorganization at the cell surface. Gelsolin is also part of the whirlin interactome and is lost from stereocilia when the critical scaffold protein whirlin is absent or when proteins such as myosin XVa are absent which are important for trafficking whirlin to the stereocilia tip.

We have shown on the basis of the knock-out phenotype that gelsolin, presumably via its capping and severing functions, acts to control actin polymerization and stereocilia development. A number of phenotypes were previously detected in the gelsolin knock-out including platelet shape changes leading to prolonged bleeding times and defective neutrophil migration *in vitro* [15]. Dermal fibroblasts derived from gelsolin mutant mice have excessive actin stress fibers and migrate slower than wild-type fibroblasts, but have increased contractility in vitro. Overall, the phenotypic analysis of the knockout indicates that gelsolin is important for rapid motile responses in cell types involved in stress responses such as hemostasis, inflammation, and wound healing. The studies reported here reveal a novel function for gelsolin involved with actin regulation in stereocilia in hair cells.

PIP_2_ plays an important role in the regulation of gelsolin function by binding to gelsolin and inhibiting its interaction with actin. PIP_2_ and other phosphoinositides may play a role in dissociating gelsolin from the plus end of actin filaments and initiating actin elongation and therefore may function in stereocilia growth. PIP_2_ regulates TRP channels and PIP_2_ depletion in hair cells leads to loss of mechanotransduction and adaptation [20]. PIP2 is detected throughout the hair cell's basolateral membrane but is absent from the apical cell surface and the taper region of the stereocilia [20]. It is however present in stereocilia above the ankle link region where its distribution would clearly overlap with gelsolin. It has been surmised that it is important to maintain PIP_2_ domains in stereocilia for controlling the turnover of actin filaments [20]. Control of actin polymerisation could be exerted through the regulation of gelsolin and its activities in capping and severing actin filaments.

Intriguingly, in the gelsolin mutant defects in stereocilia are confined to the apical regions of the cochlea. In rodents and birds, stereocilia length varies according to position in the cochlea [21], [22]. For example, in hamsters stereocilia length increases gradually from the base to apex of the cochlea [22]. Taken together, these observations may reflect a diversity of regulatory proteins interacting with the whirlin complex or other protein complexes involved with stereocilia elongation, that regulate stereocilia growth across distinct but potentially overlapping domains of the cochlea. It is noteworthy that, though the emergence of the mutant phenotype (at P9) overlaps the period in which gelsolin is expressed, stereocilia defects appear relatively late despite a complete absence of gelsolin in the developing hair bundle. The evidence that a number of regulatory proteins must be involved with the control of stereocilia growth may underlie the lag period that is seen between the absence of gelsolin and the appearance of the phenotype. It has recently been reported that twinfilin 2, an actin capping protein, is found at the tips of the short and middle rows of stereocilia from postnatal day 5 onwards at the time at which these stereocilia rows stop growing [23]. The localisation of twinfilin 2 in part mimics that of gelsolin in that the protein is not displayed in the tallest stereocilia row. Overexpression of twinfilin 2 leads to a significant reduction in the length of the tallest stereocilia in the bundle, suggesting as with gelsolin that it plays a role in regulating the length of stereocilia. That tall stereocilia are affected by twinfilin 2 overexpression, despite being confined normally to the shorter stereocilia, has been suggested to arise due to a number of reasons [23]. The shortening of the tall stereocilia could result from high levels of twinfilin 2 expression as well as immature gate-keeping at the stereocilia base, or alternatively reflect that only the tall stereocilia grow during the period investigated. Moreover, it has also been reported that myosin VIIA interacts with twinfilin 2 [24]. It is proposed that there may be an interplay between the myosin VIIA complex and the whirlin complex that plays a role in determining the staircase pattern of stereocilia within the stereocilia bundle, but this remains to be investigated. In conclusion it will be important to investigate the relative contributions of gelsolin and twinfilin 2 to stereocilia elongation, and in particular to investigate potentially overlapping domains of function by the analysis of a twinfilin 2 mutant, and to explore the interactions between the two proteins in a double mutant.

Gelsolin is also not found in inner hair cells, similar to p55, and it would seem that a completely different set of proteins scaffolded by whirlin must act to control inner hair cell stereocilia elongation. In summary, our observations on the phenotype in the gelsolin mutant indicate that the mechanisms controlling stereocilia elongation are probably diverse involving several regulatory proteins. It will be important to ascertain the localisation and function of other actin regulating molecules in other cochlear regions. The identification of gelsolin provides for the first time a link between the whirlin scaffolding protein complex involved in stereocilia elongation and a known actin regulatory molecule.

## Materials and Methods

### Ethics Statement

All animal studies were licensed by the U.K. Home Office under the Animals (Scientific Procedures) Act 1986 and were approved by Medical Research Council Harwell Ethical Review Committee.

### Mouse breeding

Gelsolin heterozygous mice (*Gsn^tm1Djk^/+*) on a mixed C57BL/6, BALB/c, 129/Sv background generated as described [15] were obtained from the MMRC (Mutant Mouse Regional Resource Centre: Stock 000133-UNC). Heterozygotes were bred to generate homozygous null (*Gsn^tm1Djk^/Gsn^tm1Djk^*), heterozygote (*Gsn^tm1Djk^/+*) and wild-type (*+/+*) littermates for both SEM and ABR analysis (see below).

### *In vitro* pull down assays

The *in vitro* pull down assays were carried out using the Promega MagneGST Pull-Down System according to the manufacturer's instructions. Briefly the C-terminal region (Lys323 through Tyr466) including the guanylate kinase (GUK) domain of p55 was radiolabelled with ^35^S by *in vitro* translation using the T7-coupled transcription-translation system (Promega). GST-tagged gelsolin constructs containing either S1 and S2 (P1), S3 and S4 (P2) or S5 and S6 (P3) gelsolin domains were expressed in E. coli K12 ER2508 cells using pGEX-4T1. Immobilised protein was then incubated with the *in vitro* translated ^35^S-labelled GUK domain of p55. GST protein alone was used as a negative control. Bound proteins were analysed on 12.5% SDS-polyacrylamide gels that were then dried and autoradiographed.

### Immunoprecipitation

Cochleas from wild type mice aged between P7 and P8 were homogenised in NP40 Lysis buffer (150 mM NaCl, 1% NP40, 50 mM Tris pH 8.0) plus protease inhibitors (Roche). The homogenate was centrifuged (20 min, 13000 g) and the supernatant used for immunoprecipitation. 6 mg total protein was precleared with 70 µl bed volume Protein G-agarose beads (Sigma) in a total volume of 500 µl for 30 minutes at 4°C. Precleared lysate was split into two and each aliquot incubated with either 50 µg of p55 antibody linked to agarose beads (Santa Cruz sc-13603 AC) or 50 µg of normal goat IgG linked to agarose beads (Santa Cruz sc-2346) as a negative control, in a total volume of 500 µl, overnight at 4°C. The beads were then washed three times with 500 µl NP40 Lysis buffer plus a protease inhibitor cocktail. Immunoprecipitated proteins were eluted in 0.1 M glycine pH 2 and immediately neutralized with 1 M Tris base. A sample of the eluted proteins was resolved in a 4–12% NuPAGE gel (Invitrogen) and Coomassie blue stained. The remaining elution was analysed by LC-MALDI TOF/TOF.

### Antibodies and Immunohistochemistry

Goat anti-p55 (T-19) and anti-gelsolin (N-18) were purchased from Santa Cruz Biotechnology, Inc. Whole-mount immunostaining for mouse cochlea was performed as described (Kikkawa et al. 2005).

### Scanning Electron Microscopy

Freshly dissected inner ears were fixed for 3 to 4 hours in 2.5% gluteraldehyde in 0.1 M phosphate buffer pH 7.3 at 4°C. After 3 washes of 15 minutes in 0.1 M phosphate buffer, the ears were decalcified in 4.3% EDTA in 0.1 M phosphate buffer pH 7.3 for 48 hours after which they were dissected to expose the organ of Corti. The ears were then dehydrated in ethanol, critical point dried, sputter coated with gold and viewed on a Hitachi S530 Scanning Electron microscope.

### Auditory-evoked brainstem response analysis (ABR)

Mice were anaesthetised with ketamine (Ketaset™) and additionally given medetomidine (Domitor™) for muscle relaxation and analgesia by intraperitoneal injection (0.5 ml Domitor™ at 100 mg/ml with 4.12 ml water and 0.38 ml Ketaset™ at 1 mg/ml; administered at a rate of 0.1 ml/10 g of body weight). Animals were placed in an audiometric chamber (IAC 401-A-SE) on a heated mat to maintain body temperature. Acoustic stimuli were delivered monaurally to the right ear at a distance of 1.5 cm via a free field transducer (ES1 Tucker Davis Technology (TDT), Alachua, FL), controlled by SigGen/BioSig software (TDT), using TDT system III hardware. The transducer was calibrated using a ¼” measuring microphone (7016 ACO-Pacific, Belmont, CA) and SigCal software (TDT). Electrodes (Grass Telefactor F-E2-12) were placed sub-dermally over the vertex (active), right mastoid (reference), and left mastoid (ground). The responses from the electrodes were amplified using TDT system III hardware and averaged using BioSig software. Click stimuli consisted of a 0.1 ms broadband click of alternating polarity. Tone-burst stimuli totalled 7 ms duration including 1 ms rise/fall time. Both were presented at 90dB SPL followed by decreasing steps of 10dB, until a threshold was reached. For tone-burst the frequencies used were 4, 8, 16, 26 and 32 kHz. Recovery of mice was accelerated by administration of atipamezole (Antisedan™, 5 mg/ml) at the rate of 1 mg/kg of body weight.

## Supporting Information

Figure S1 Ultrastructural analysis of stereocilia of basal cochlear outer hair cells and cochlear inner hair cells in gelsolin mutant mice. a. Analysis of outer hair cell morphology in the basal turns in wild-type and gelsolin homozygous knock-out mice (Gsntm1Djk/Gsntm1Djk). Bundle morphology in the Gsntm1Djk/Gsntm1Djk mice at the basal turn is normal. (Scale bar, 10 µm). b. Analysis of inner hair cell morphology along the cochlear turn in wild-type and gelsolin homozygous knock-out mice (Gsntm1Djk/Gsntm1Djk). Bundle morphology in the Gsntm1Djk/Gsntm1Djk mice is normal in inner hair cells. (Scale bar, 10 µm).
(1.03 MB TIF)

Figure S2 Analysis of Vestibular Hair cells of gelsolin mutant mice a. Gelsolin is not expressed in vestibular hair cells. Vestibular whole mounts from P13 mice utricle were stained with antibody to gelsolin (green) and phalloidin (red) to detect actin. b. Analysis of vestibular hair cell morphology in wild-type and gelsolin homozygous knock-out mice (Gsntm1Djk/Gsntm1Djk). Bundle morphology in the Gsntm1Djk/Gsntm1Djk mice is normal in vestibular hair cells. (Scale bar, 20 µm)
(0.50 MB TIF)

Figure S3 Auditory brainstem response (ABR) analysis in 2 month old gelsolin mutant mice. An audiogram plotting the average ABR thresholds from Gsntm1Djk/Gsntm1Djk (HOM; n = 3) and +/+ sibs (WT; n = 3) mice at 8 weeks of age. The audiogram shows there were no significant differences between any of the genotypes at any of the test frequencies 4, 8, 16, 26 and 32 kHz. The error bars show the standard error of the mean. The poor thresholds observed at 32kHz in both wild-type and mutant mice may partly reflect the presence of the ahl 753A susceptibility allele at the Cdh23 locus which is present in the background strains of the gelsolin knock-out mouse line [Noben-Trauth K, Zheng QY, Johnson KR (2003) Association of cadherin 23 with polygenic inheritance and genetic modification of sensorineural hearing loss. Nature Genetics 35: 21–23].
(0.19 MB TIF)
