# Supplementary figures and images for: Gelsolin Plays a Role in the Actin Polymerization Complex of Hair Cell Stereocilia
Source: PLoS One. 2010 Jul 16;5(7):e11627. doi: 10.1371/journal.pone.0011627 (PMC2905391; doi:10.1371/journal.pone.0011627)

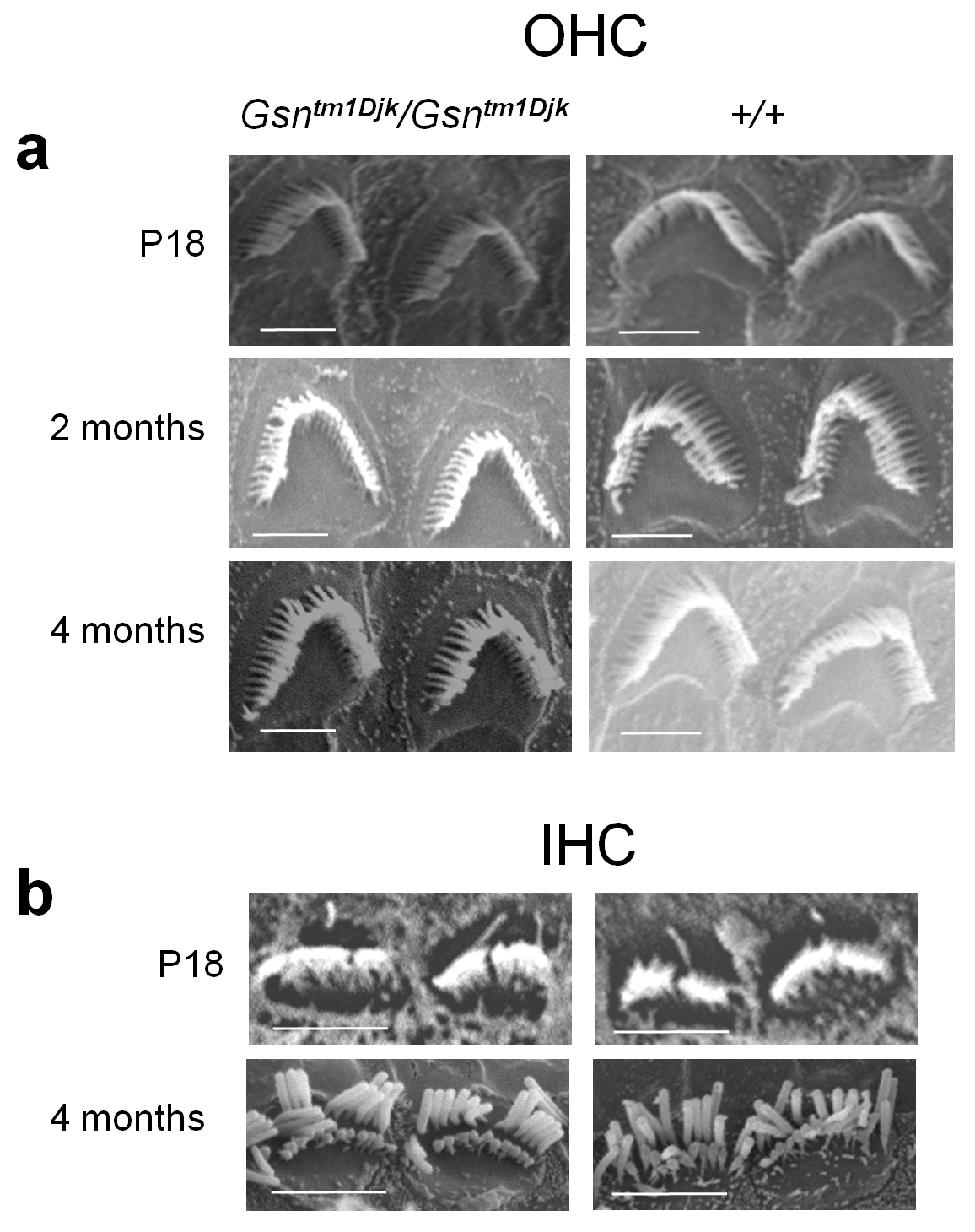

Supplement: Figure S1 — Ultrastructural analysis of stereocilia of basal cochlear outer hair cells and cochlear inner hair cells in gelsolin mutant mice. a. Analysis of outer hair cell morphology in the basal turns in wild-type and gelsolin homozygous knock-out mice (Gsntm1Djk/Gsntm1Djk). Bundle morphology in the Gsntm1Djk/Gsntm1Djk mice at the basal turn is normal. (Scale bar, 10 µm). b. Analysis of inner hair cell morphology along the cochlear turn in wild-type and gelsolin homozygous knock-out mice (Gsntm1Djk/Gsntm1Djk). Bundle morphology in the Gsntm1Djk/Gsntm1Djk mice is normal in inner hair cells. (Scale bar, 10 µm). (1.03 MB TIF) [file pone.0011627.s001.tif]

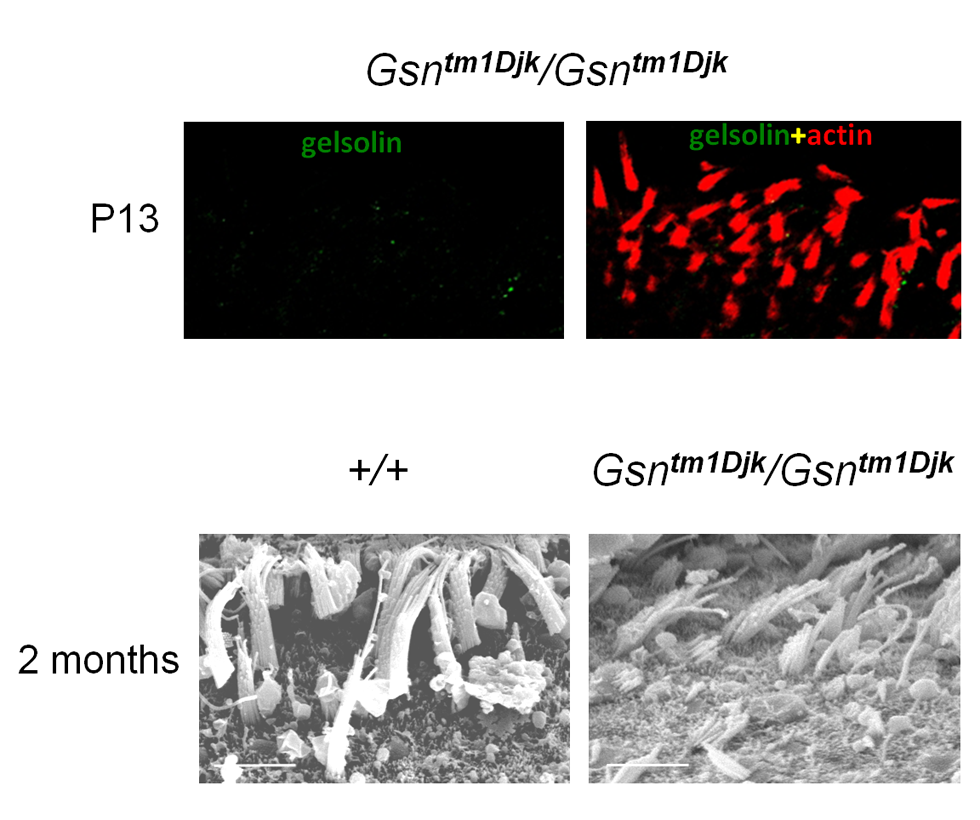

Supplement: Figure S2 — Analysis of Vestibular Hair cells of gelsolin mutant mice a. Gelsolin is not expressed in vestibular hair cells. Vestibular whole mounts from P13 mice utricle were stained with antibody to gelsolin (green) and phalloidin (red) to detect actin. b. Analysis of vestibular hair cell morphology in wild-type and gelsolin homozygous knock-out mice (Gsntm1Djk/Gsntm1Djk). Bundle morphology in the Gsntm1Djk/Gsntm1Djk mice is normal in vestibular hair cells. (Scale bar, 20 µm) (0.50 MB TIF) [file pone.0011627.s002.tif]

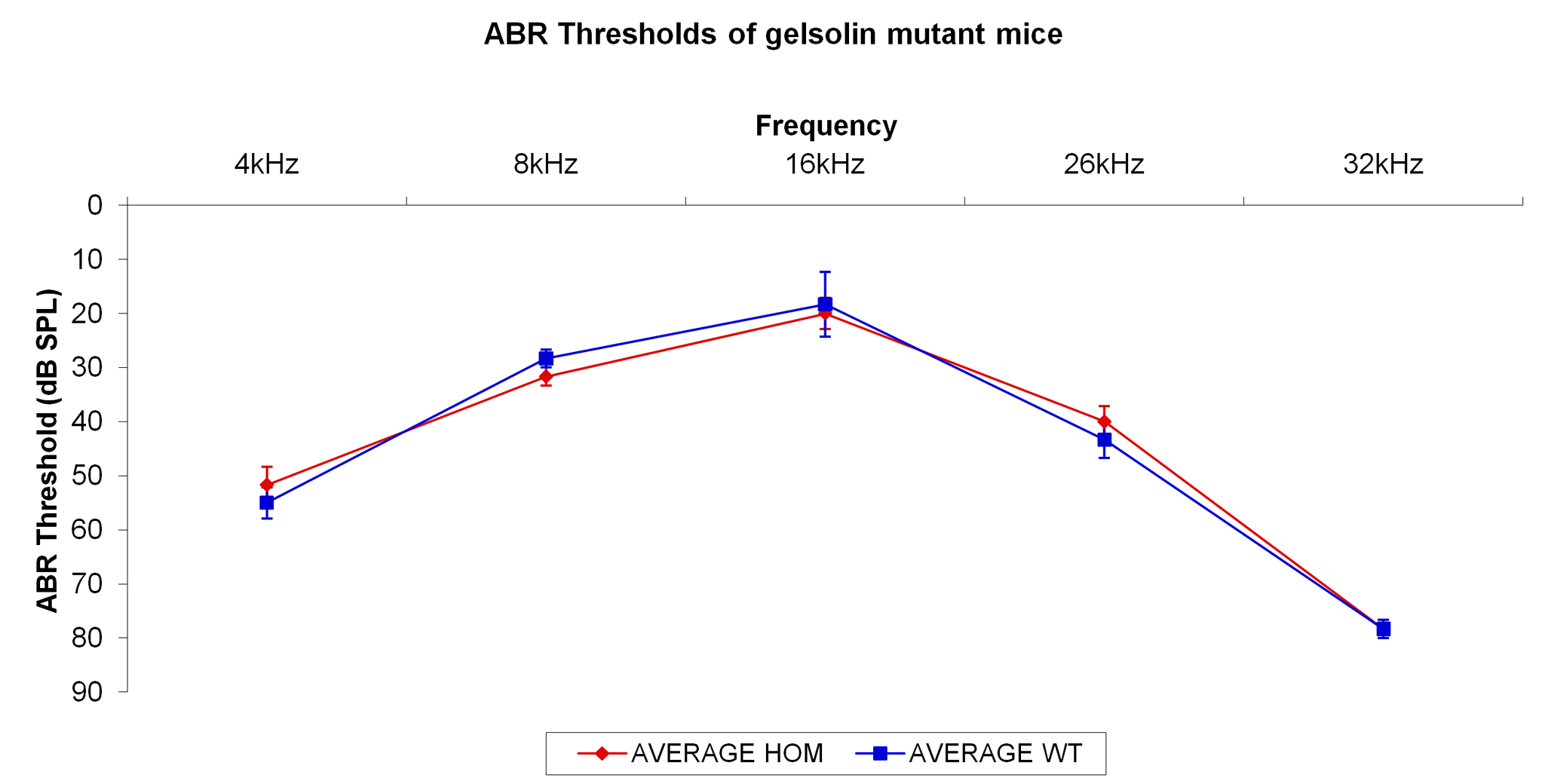

Supplement: Figure S3 — Auditory brainstem response (ABR) analysis in 2 month old gelsolin mutant mice. An audiogram plotting the average ABR thresholds from Gsntm1Djk/Gsntm1Djk (HOM; n = 3) and +/+ sibs (WT; n = 3) mice at 8 weeks of age. The audiogram shows there were no significant differences between any of the genotypes at any of the test frequencies 4, 8, 16, 26 and 32 kHz. The error bars show the standard error of the mean. The poor thresholds observed at 32kHz in both wild-type and mutant mice may partly reflect the presence of the ahl 753A susceptibility allele at the Cdh23 locus which is present in the background strains of the gelsolin knock-out mouse line [Noben-Trauth K, Zheng QY, Johnson KR (2003) Association of cadherin 23 with polygenic inheritance and genetic modification of sensorineural hearing loss. Nature Genetics 35: 21–23]. (0.19 MB TIF) [file pone.0011627.s003.tif]
